# Genomic methylation patterns in pre-meiotic gynoecia of wild-type and RdDM mutants of Arabidopsis

**DOI:** 10.3389/fpls.2023.1123211

**Published:** 2023-03-13

**Authors:** Quetzely Ortiz-Vasquez, Gloria León-Martínez, Carlos Barragán-Rosillo, Eduardo González-Orozco, Samuel Deans, Billy Aldridge, Martin Vickers, Xiaoqi Feng, Jean-Philippe Vielle-Calzada

**Affiliations:** ^1^ Grupo de Desarrollo Reproductivo y Apomixis, Unidad de Genómica Avanzada Laboratorio Nacional de Genómica para la Biodiversidad, CINVESTAV, Irapuato, Guanajuato, Mexico; ^2^ Department of Cell and Developmental Biology, John Innes Centre, Norwich, United Kingdom

**Keywords:** DNA methylation, RNA directed DNA methylation (RdDM), ARGONAUTE, gynoecium, reproduction

## Abstract

**Introduction:**

Although DNA methylation patterns are generally considered to be faithfully inherited in Arabidopsis thaliana (Arabidopsis), there is evidence of reprogramming during both male and female gametogenesis. The gynoecium is the floral reproductive organ from which the ovules develop and generate meiotically derived cells that give rise to the female gametophyte. It is not known whether the gynoecium can condition genomic methylation in the ovule or the developing female gametophyte.

**Methods:**

We performed whole genome bisulfite sequencing to characterize the methylation patterns that prevail in the genomic DNA of pre-meiotic gynoecia of wild-type and three mutants defective in genes of the RNA-directed DNA methylation pathway (RdDM): ARGONAUTE4 (AGO4), ARGONAUTE9 (AGO9), and RNA-DEPENDENT RNA POLYMERASE6 (RDR6).

**Results:**

By globally analyzing transposable elements (TEs) and genes located across the Arabidopsis genome, we show that DNA methylation levels are similar to those of gametophytic cells rather than those of sporophytic organs such as seedlings and rosette leaves. We show that none of the mutations completely abolishes RdDM, suggesting strong redundancy within the methylation pathways. Among all, ago4 mutation has the strongest effect on RdDM, causing more CHH hypomethylation than ago9 and rdr6. We identify 22 genes whose DNA methylation is significantly reduced in ago4, ago9 and rdr6 mutants, revealing potential targets regulated by the RdDM pathway in premeiotic gyneocia.

**Discussion:**

Our results indicate that drastic changes in methylation levels in all three contexts occur in female reproductive organs at the sporophytic level, prior to the alternation of generations within the ovule primordium, offering a possibility to start identifying the function of specific genes acting in the establishment of the female gametophytic phase of the Arabidopsis life cycle.

## Introduction

Unlike animals, plant gametic precursor cells are not determined during early embryogenesis but during flower development (Maine, 2013). In Arabidopsis, the gynoecium is located in the innermost whorl of the flower and corresponds to the female reproductive organ (Herrera-Ubaldo and de Folter, 2018), and formed after the emergence of two distinct carpels from the floral meristem. Their fusion during early differentiation progressively gives rise to a cylindrical organ composed of lateral and medial domains that will form the valves and replum, respectively. Within the growing cylinder, an internal carpel margin meristem will differentiate 50 to 60 ovule primordia that will undergo meiosis and female gametophyte development before pollination (Huang et al., 2013; Herrera-Ubaldo and de Folter, 2022). The differentiation of a single meiotic precursor - the megaspore mother cell (MMC) - marks the transition from somatic to reproductive fate and is essential for the initiation of the female gametophytic phase of the Arabidopsis life cycle. After undergoing meiosis, the MMC gives rise to four haploid cells, with only one surviving to undergo three rounds of mitotic divisions before cellularization and formation of the female gametophyte (Lieber et al., 2011).

Several reports have identified that small RNAs (sRNAs), DNA methylation, chromatin remodeling, and phytohormone signaling play an important role in the specification of the female gametophytic lineage of flowering plants (García-Aguilar et al., 2010; Olmedo-Monfil et al., 2010; Singh et al., 2011; Su et al., 2017; Su et al., 2020; Cai et al., 2022). It is generally considered that one of the main purposes of DNA methylation is to control the integrity of the genome by preventing the expression and mobility of transposable elements (TEs) and allowing monoallelic expression of maternal or paternal genes in the endosperm (Cao et al., 2000; Vigneau and Borg, 2021). More recently, DNA methylation has been associated with a role in regulating the efficiency of mRNA splicing (Lev-Maor et al., 2015; Wang et al., 2016; Walker et al., 2018) and avoiding transcription from cryptic transcriptional sites within genes (Choi et al., 2020; Le et al., 2020).

DNA methylation occurs in CG, CHG, CHH contexts (where H can be adenine, cytosine, or thymine). Whereas *de novo* methylation refers to process by which previously unmethylated cytosine residues are methylated - resulting in the formation of new methylation patterns -, maintenance methylation relies on pre-existing methylation marks to guide their methylation during replication (Pavlopoulou and Kossida, 2007). DNA *de novo* methylation in either CG, CHG or CHH context is established by the RNA-directed DNA methylation pathway (RdDM) (Law and Jacobsen, 2010; Matzke and Mosher, 2014; Erdmann and Picard, 2020). Maintenance of CG methylation is carried by METHYLTRANSFERASE1 (MET1) (Kankel et al., 2003), whereas CHG methylation is dependent on the CHROMOMETHYLASE3 (CMT3) pathway (Lindroth et al., 2001). CHH methylation is maintained by both the RdDM and the CHROMOMETHYLASE2 (CMT2) pathway, mostly in non-overlapping functional contexts. RdDM is known to be an important regulator of widely diverse plant mechanisms that include response to abiotic stress and pathogens, cell to cell signaling, transposon silencing, genome stability, and reproductive development (Erdmann and Picard, 2020; Kumar et al., 2021). RdDM occurs in euchromatin regions of genes and in short TEs distributed along chromosomes, especially in heterochromatic regions (Durán-Figueroa and Vielle-Calzada, 2010; Matzke and Mosher, 2014; Erdmann and Picard, 2020; Long et al., 2021). In contrast, CMT2-dependent methylation occurs preferentially in heterochromatin and the long TEs, as well as centromeric and pericentromeric regions (Zemach et al., 2013; Stroud et al., 2014; He et al., 2021).

The RdDM pathway has canonical and non-canonical mechanisms that are functionally similar but not equivalent. After cleavage of transcribed non-coding RNA precursors, single-stranded sRNAs are incorporated into an ARGONAUTE (AGO) protein of the AGO4 clade (AGO4, AGO6, AGO8, or AGO9) and directed towards complementary PolV-derived chromatin-bound transcripts, before getting incorporated into a chromatin-binding complex involving RNA POLYMERASE V (PolV) and DOMAINS REARRANGED METHYLTRANSFERASE2 (DRM2) (Cuerda-Gil and Slotkin, 2016). Whereas canonical RdDM depends on RNA-DEPENDENT RNA POLYMERASE2 for double stranded sRNA biogenesis, non-canonical RdDM pathways rely on RNA-DEPENDENT RNA POLYMERASE6 (RDR6) for the equivalent process, an enzyme also important for the silencing of transcriptionally active TEs (Cuerda-Gil and Slotkin, 2016).

Mutations in multiple genes involved in the RdDM pathway show the differentiation of ectopic female gametic precursors in the pre-meiotic ovule of Arabidopsis (Olmedo-Monfil et al., 2010; Hernández-Lagana et al., 2016). Unlike recessive mutations in the *RETINOBLASTOMA RELATED* (*RBR*) gene, ectopic cells adjacent to the MMC in all dominant mutants of the AGO4 clade (*ago4, ago6, ago8*, and *ago9*) – as well as mutations in *RDR6, DICER LIKE3*, and *POLIV POLV* - undergo mitosis rather than meiosis (Olmedo-Monfil et al., 2010), a phenomenon that is reminiscent to apospory in flowering plants. *AGO9* is expressed in developing ovules and the female gametophytes, particularly in the L1 epidermal layer of ovule primordia prior to meiosis, but also in anthers and developing pollen grains, as well as meristematic cells (Olmedo-Monfil et al., 2010; Ashapkin et al., 2019; Gutzat et al., 2020). *AGO9* expression can restore DNA methylation in an *ago4* mutant if expressed from the *AGO4* promoter (Havecker et al., 2010), suggesting functional complementarity among AGO4-clade proteins (Zilberman et al., 2003; Havecker et al., 2010; Matzke and Mosher, 2014). Immunoprecipitation-based protein analysis suggests that AGO9 binds to DRM2, and genome-wide association studies indicate that perhaps also with CMT2 (Zhong et al., 2014; Kawakatsu et al., 2016), suggesting a wide functional versatility and RdDM related function during reproductive development.

To establish the role of the RdDM pathway during female reproductive development, we used whole genome bisulfite sequencing to characterize the methylation patterns that prevail in gynoecia containing premeiotic ovules of wild-type plants, as well as *ago4*, *ago9* and *rdr6* homozygous individuals. After confirming biological reproducibility of the methylation patterns across the genome, we demonstrated that DNA methylation levels in TEs and genes in wild-type pre-meiotic gynoecia are similar to those of gametophytic cells rather than those of sporophytic tissues such as seedlings and rosette leaves. We also show that loss of genomic methylation in pre-meiotic gynoecia of *ago4*, *ago9*, and *rdr6* is not prevalent in all three contexts, as *AGO9* and *RDR6* preferentially tend to repress methylation in the CHH context. We provide a compendium of genomic regions targeted by *AGO4*, *AGO9*, and *RDR6*, showing that these three genes affect methylation at different loci. We also confirm that they mainly participate in the methylation of TEs in a CHH context, identifying a group of 22 genes that are redundantly methylated by their common action. Our results indicate that drastic changes in methylation levels for both CHH and CHG occur in female reproductive organs at the sporophytic level, prior to the alternation of generations within the ovule primordium, offering a possibility to start identifying the function of specific changes in genomic methylation patterns during female reproductive development.

## Materials and methods

### Plant materials

Seeds from wild-type and insertional *ago4-6* (SALK_071772), *ago9-3* (SAIL_34_G10), *rdr6-15* (SAIL_617) mutants (all in a *Col*-0 background) were disinfected with 40% chlorine for 10 minutes under constant agitation, rinsed three times with sterile distilled water, planted in petri plates containing 0.5% Murashige and Skoog (MS) medium, vernalized for three days at 4°C under dark conditions, and germinated at 21°C in long daylight conditions (16h light/8h dark). Seedlings were transferred to soil (3:1:1 Sunshine substrate: vermiculite: perlite), grown under greenhouse conditions and fertilized with slow release 14-14-14 fertilizer (1.84kg/m^3^). Homozygous *ago9* and *rdr6* individuals were identified by PCR genotyping using primer combinations listed in **Supplementary Table S1**. In the case of *rdr6-15*, amplified DNA products were digested with MscI (New England Biolabs, USA) to distinguish wild-type and mutant alleles.

### DNA extraction and whole genome bisulfite sequencing

For each biological replicate, 200 gynoecia of precisely 0.5 mm in length – and containing ovules at stages 1 and 2 (Rodríguez-Leal et al., 2015) - were isolated and collected under a Leica M80 stereomicroscope. Stage 9 and 10 flower buds (Schneitz et al., 1995) were isolated using emasculation tweezers (Dumont #5 Tweezers; Electron Microscopy Sciences) and immobilized on a slide with double-sided adhesive tape (**Supplementary Figure S1**). Gynoecia measuring 0.5 mm were isolated and collected using 1 ml insulin syringes (Terumo, USA), immediately placed 1.5 ml microcentrifuge tubes in liquid nitrogen, and stored at -80°C before DNA extraction.

DNA extraction was performed with cetyltrimethyl ammonium bromide (CTAB) buffer adding a final incubation step with RNase (modified CTAB protocol on the base of Doyle and Doyle, 1987). For bisulfite conversion of unmethylated cytosines to uracil, we used the EpiTect Fast Bisulfite Conversion kit (Qiagen, #59802). Library construction was performed using the Ovation Ultralow Methyl-Seq Library System kit (Nugen, #0336), following two rounds of bisulfite conversion (Walker et al., 2018). Two biological replicates were generated for each genotype. Wild-type replicate #1 (Wt_R1), *ago4* replicate #1 (*ago4_*R1), *ago9* replicate #1 *(ago9_*R1), and *ago9* replicate #2 *(ago9_*R2) libraries were generated from 59 to 120 ng of total DNA that was treated with bisulfite as described above, and subsequently amplified with 10 cycles of PCR. Wild-type replicate #2 (Wt_R2) and *ago4* replicate #2 (*ago4*_R2) libraries were generated from 70 ng of total DNA treated with bisulfite and subsequently amplified with 11 cycles of PCR. Finally, *rdr6* replicate #1 (*rdr6*_R1) and *rdr6* replicate #2 (*rdr6*_R2) were generated from 20 and 29 ng of total DNA, and amplified with 13 and 12 cycles of PCR, respectively. All libraries were sequenced using the single-end Illumina HiSeq mode.

### Quality read identification and analysis of methylation levels

Library adapters, the initial nine nucleotides (nt) from the 5’ end, short sequences of less than 20 nt, and low-quality reads (Phred score less than 20) were discarded from all methylomes using the TrimGalore version 0.4.2 (Krueger F. https://github.com/FelixKrueger/TrimGalore). Resulting reads were mapped to the Arabidopsis TAIR10 genome after *in silico* converting all sense cytosines to thymine, and all antisense guanines to adenine, allowing one mismatch per sequence and keeping only reads showing a single best alignment when using the Bismark version v0.19.0 (Krueger and Andrews, 2011). Duplicate reads were removed using the Samtools version 1.9, rmdups option (Danecek et al., 2021). Reads that showed a perfect match to the unconverted TAIR10 genome were eliminated by considering they represent DNA residues unaffected by bisulfite reactions (less than 1.1% of all sequences in all cases). In each of the eight resulting methylomes (two replicates per genotype), the total number of methylated and unmethylated cytosines were determined in each context (CG, CHH, and CHG) using the Bismark-methylation-extractor tool (Krueger and Andrews, 2011) in both sense and antisense genomic strands. The single nucleotide methylation frequency was calculated by estimating the number of cytosines over the number of cytosines and thymines at each individual genomic site. Genomic bins of 50 nt were considered to estimate the methylation frequency corresponding to the ratio of the total number of methylated cytosines by the total number of cytosines included in each bin.

### Methylation profiles and differentially methylated regions

Genes and transposable elements (TEs) annotated in TAIR10 were aligned at the 5’ end or the 3’ end, discarding from the analysis either 1500 bp (genes) or 250 bp (TEs), from the end opposite to the one used for alignment (Ibarra et al., 2012). Methylation levels were averaged across 100 bp intervals using the number of cytosines and thymines sequenced at the single nucleotide level for each sequence context in a given methylome. Finally, average methylation levels were calculated over a region encompassing 5 kb upstream and 5 kb downstream of the alignment site. Resulting methylation profiles were compared to available datasets for seedling (Zhang et al., 2013), rosette leaf (Stroud et al., 2014), central cell (Park et al., 2016), male meiocyte (Walker et al., 2018), and ovule (Zhou et al., 2022).

A potential differentially methylated region (DMR) was defined as a genomic segment with a minimum length of 100 nt that contained at least 20 sites corresponding to cytosines, and showed at least 10% (CHH context), 20% (CHG context) or 40% (CG context) methylation difference between wild-type and a specific RdDM mutant. Potential DMRs of less than 100 nt were discarded. The 100 nt window is a standard arbitrary parameter that accounts for frequent limited cytosine coverage in 50 nt genomic windows, increasing the probability of detecting significant methylation differences over a larger window, but maintaining the length within the range of a single nucleosome to prevent uncertain biological interpretations. Two 50 nt windows separated by more than 100 nt were considered to represent independent DMRs. The statistical value of all DMRs was determined by applying a Fisher’s exact test with a p-value of less than 0.001. DMRs were considered to map to a promoter, gene body, or TEs, when at least 50% of the DMR overlapped with either 200 bp upstream of the first exon (promoter), with a region encompassing the first and last exon of an annotated transcriptional unit (gene), or with 50% of a previously annotated TE. DMRs were considered to map to gene elements if 50% of their length overlapped with a gene unit, considering 200 bp upstream of the first exon as part of the gene unit.

## Results

### Methylation levels in pre-meiotic gynoecia reveal unexpected similarity to gametophytic cells

We confirmed the reproducibility of biological replicates for all four genotypes by conducting a principal component analysis (PCA) using cytosine sites in a CG context that were covered in all eight methylomes (two biological replicates per genotype), for a total of 4.1x10^5^ sites. As illustrated in **Supplementary Figure S2**, components 1 and 2 explain 27% and 25% of the variation between replicates, respectively. All samples had a reproducible tendency two group within their corresponding genotype (wild-type, *ago4*, *ago9*, or *rdr6*), with wild-type and *ago9* methylomes showing less variability than methylomes of *ago4* and *rdr6*.

**Figure 1:**
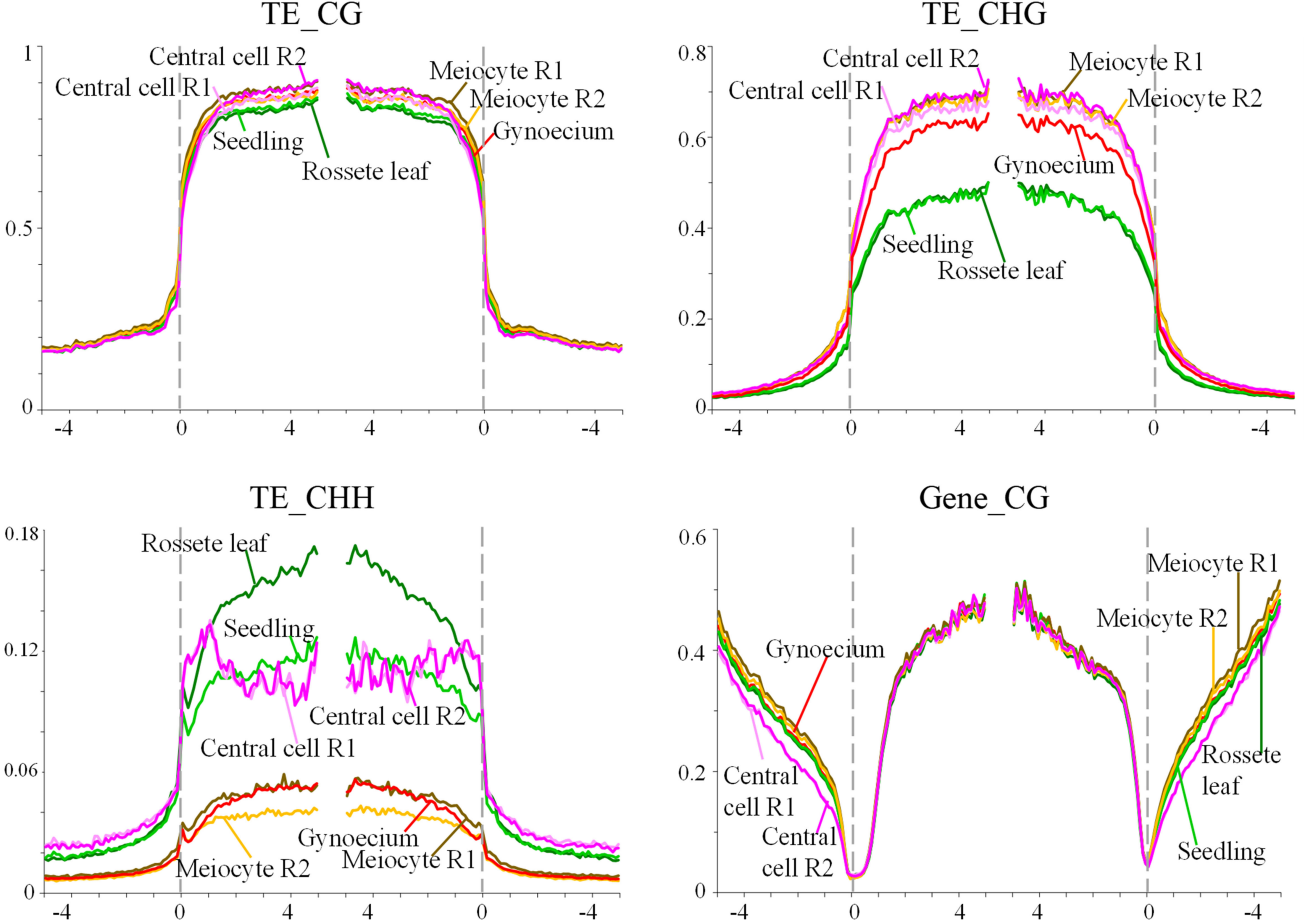
Methylation levels of TEs and genes in the CHH and CG contexts. The graph shows the methylation frequency shown at each nucleotide position for alignments in all three contexts (TEs), or the CG context (genes); rosette leaf (dark green), seedling (light green), central cell replicate #1 (Central cell R1) (light pink), central cell replicate #2 (Central cell R2) (dark pink), male meiocyte replicate #1 (Meiocyte R1) (brown), male meiocyte replicate #2 (Meiocyte R2) (yellow), wild-type gynoecium (red).

For genes and TEs, we compared the global methylation profile of the pre-meiotic wild-type gynoecium to methylation profiles from other sporophytic organs or gametophytic cells. These included seedlings (Zhang et al., 2013), rosette leaves (Stroud et al., 2014), the central cell of the female gametophyte (Park et al., 2016), and the male meiocyte (Walker et al., 2018). These results are illustrated in **Figure 1**. For TEs, wild-type methylation levels in the CG context were higher in the central cell and the male meiocyte than in rosette leaves or seedlings, consistent with the prior knowledge that reproductive cells have higher CG methylation than somatic tissues (Hsieh et al., 2016; Park et al., 2016). Strikingly, wild-type TEs methylation levels in the CG context of pre-meiotic gynoecia were higher than expected, reminiscent of those prevailing in the male meiocyte rather than those of sporophytic organs (**Figure 1**). Methylation levels of TEs in the CHG context were also similar to gametophytic cells, significantly higher than those of sporophytic organs. And for the CHH context, TE methylation levels in pre-meiotic gynoecia are also similar to those of the male meiocyte, but lower than those found in seedlings, rosette leaves and the central cell. In the case of genes in the CG context, methylation levels were quite similar for all organs and cells analyzed (**Figure 1**). The overall methylation levels for TEs and genes had little difference from wild type in pre-meiotic gynoecia of homozygous *ago4*, *ago9*, and *rdr6* individuals, suggesting that they are not majorly involved in the establishment of high methylation levels observed in TEs and gene bodies (**Supplementary Figure S3**).

To determine if these results reveal a tendency to establish gametophytic methylation levels early during female reproductive organ development, we compared methylation levels of TEs and genes in pre-meiotic gynoecia and ovules, using a recently available dataset generated by Zhou et al. (2022). As illustrated in **Supplementary Figure S4**, methylation levels in TEs and genes are similar in both organs, in both cases reminiscent of those prevailing in male meiocytes, confirming that drastic changes in methylation levels for CHH, CG and CHG contexts occur at the sporophytic level, and prior to the alternation of generations within the ovule primordium.

### Loss of genomic methylation in pre-meiotic gynoecia of RdDM mutants is not prevalent in all three contexts

The wild-type pre-meiotic gynoecia is characterized by overall levels of genomic methylation that reach 22.37%, 8.63%, and 1.14% for the CG, CHG, and CHH context, respectively (**Supplementary Table S2** **)**. As expected, the pre-meiotic gynoecium of mutants affected in the RdDM pathway show global methylation levels lower than those found in wild-type gynoecia. As expected, in all three mutants analyzed, the most drastic reduction was in the CHH context, with 34.44%, 40.7% and 35.17% less overall methylation for *ago4, ago9*, and *rdr6*, respectively (**Supplementary Table S2**).

Methylomes were compared to identify DMRs corresponding to genomic segments with a minimum length of 100 nt, between mutant and wild-type. The total number of DMRs including all those present in three different contexts was 8,417 for *ago4*, 5,127 for *ago9*, and 5,832 for *rdr6* (**Table 1**). In all three mutants, at least 90% of DMRs in the CG and CHH context had a length comprised between 100-200 nt, and between 100-250 nt for the CHG context (**Supplementary Table S3**), representing a size distribution similar to those of other mutants involved in the RdDM pathway (Yang et al., 2016). As expected for all three mutants, the DMRs in the CHH context are distributed across all 5 chromosomes, reflecting the overall genomic activity of the RdDM pathway (**Supplementary Figure S5** and **Table S4**). The proportion of DMRs corresponding to a CHH context was of 19% (*ago4*), 27.3% (*ago9*), and 26.5% (*rdr6*) for a total coverage of 227.05 kb, 188.9 kb, and 204.7 Kb, respectively (**Table 1**). This proportion is significantly smaller than DMRs found in the CHH context for other RdDM mutants such as *drm2*, *rdr2*, or mutants affecting specific subunits of *POLIV* and *POLV*, in either seedlings or the male meiocyte (Walker et al., 2018; He et al., 2021), suggesting that functional redundancy is prevalent in the pre-meiotic gynoecium to maintain DNA methylation and genome stability.

**Table 1:** Total number and length of DMRs in *ago4*, *ago9* and *rdr6*.

Sequence context	*ago4*		*ago9*		*rdr6*	
	Total DMRs	Total Kb covered	Total DMRs	Total Kb covered	Total DMRs	Total Kb covered
CG	4,416	599.05	2,337	299.15	3,038	398.05
CHG	2,395	387.40	1,388	205.55	1,245	195.55
CHH	1,606	227.05	1,402	188.95	1,549	204.75
Total	8,417	1,213.50	5,127	693.65	5,832	798.35

### *AGO4*, *AGO9* and *RDR6* participate mainly in CHH methylation of TEs

**Figure 2:**
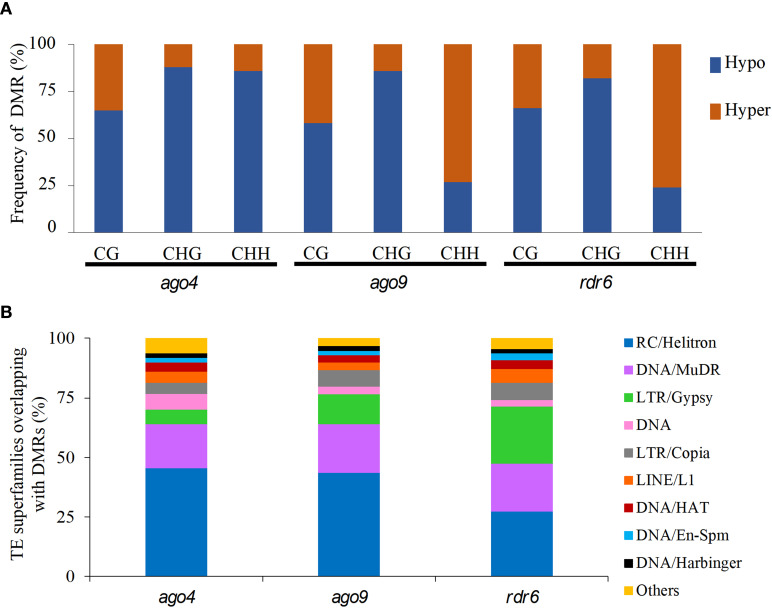
Quantification of hypo- and hypermethylated DMRs and their most represented TE superfamilies in three RdDM mutants. **(A)** Percentage of hypomethylated (Hypo) and hypermethylated (Hyper) DMRs in each mutant for each sequence context. **(B)** Representation of TE superfamilies within DMRs for all three mutants. The “Others” group includes the following TE superfamilies: DNA/Pogo, RathE1_cons, DNA/Mariner, SINE, RathE3_cons, DNA/Tc1. RathE2_cons, and SADHU.

When compared to wild-type, DMRs in *ago4*, *ago9*, and *rdr6* were most frequently hypomethylated in CG and CHG contexts (**Figure 2A**). The same is true for *ago4* in the CHH context, but not for DMRs in *ago9* and *rdr6* for the CHH context, which were preferentially hypermethylated (**Figure 2A**). Mapping of all these DMRs revealed that most of them correspond to discrete genomic regions located in TEs. The proportion of DMRs mapping to TEs corresponds to 54.4%, 65.5%, and 63.1% for *ago4*, *ago9*, and *rdr6* respectively (**Table 2** and **Supplementary Tables S5**, **S6**). As expected, the majority of DMRs map to TEs of the most abundant superfamilies, including DNA/Harbinger, LTR/Gypsy, RC/Helitron, DNA/MuDR and LTR/Gypsy, representing more than 70% of all TEs targeted by DMRs (**Figure 2B**; **Supplementary Table S5**). SINE retrotransposons represented 7.6%, 3.1%, and 1.5% of all hypomethylated TEs in *ago4*, *ago9*, and *rdr6*, respectively: most abundant hypomethylated superfamilies also included RathE1_cons and RathE2_cons in *ago4* and *ago9*; and DNA/Mariner and LRT/copy in *rdr6* (**Supplementary Figure S6** and **Supplementary Table S5**). In the case of hypermethylated DMRs, DNA/Harbinger and LTR/Gypsy TEs were among the most represented superfamilies common to all three mutants, whereas DNA/MuDR was found only in *ago4* and *ago9*, and SINE was among the most represented in *ago4* and *rdr6* (**Supplementary Figure S6** and **Supplementary Table S5**).

**Table 2:** DMRs mapping to specific genomic regions in the CHH context.

Genotype	Type of DMR	Total	Gene elements (%)	Gene promoter (%)	Gene body (%)	TEs (%)	Gene element and TEs (%)	Others (%)
*ago4*	Hypermethylated	231	57 (24.7)	6 (2.6)	51 (22.1)	155 (67.1)	15 (6.5)	34 (14.7)
	Hypomethylated	1,375	147 (10.7)	48 (3.5)	99 (7.2)	719 (52.3)	54 (3.9)	563 (40.9)
	Total	1,606	204 (12.7)	54 (3.4)	150 (9.3)	874 (54.4)	69 (4.3)	597 (37.2)
*ago9*	Hypermethylated	1023	122 (11.9)	20 (2.0)	102 (10.0)	746 (72.9)	46 (4.5)	201 (19.6)
	Hypomethylated	379	52 (13.7)	10 (2.6)	42 (11.1)	173 (45.6)	11 (2.9)	165 (43.5)
	Total	1,402	174 (12.4)	30 (2.1)	144 (10.3)	919 (65.5)	57 (4.1)	366 (26.1)
*rdr6*	Hypermethylated	1,171	103 (8.8)	21 (1.8)	82 (7.0)	789 (67.4)	35 (3.0)	314 (26.8)
	Hypomethylated	378	56 (14.8)	13 (3.4)	43 (11.4)	188 (49.7)	14 (3.7)	148 (39.2)
	Total	1,549	159 (10.3)	34 (2.2)	125 (8.1)	977 (63.1)	49 (3.2)	462 (29.8)

### *ago4*, *ago9* and *rdr6* affect methylation at distinct loci

Given that both *ago9* and *rdr6* had a higher number of hypermethylated DMRs in the CHH context and the majority mapped to TEs, we determined if families that contained more than 10% of their members targeted by hypermethylated DMRs were equivalent in *ago9* and *rdr6*. Among 54 families overall targeted by DMRs in both genes (25 in *ago9* and 29 in *rdr6*), only six were represented in hypermethylated DMRs in both mutants (**Supplementary Figure S7**), suggesting that the two corresponding genes target a distinct universe of TEs during female reproductive development. In addition, *ago4* hypermethylated TE in *ago9* and *rdr6*, with only one of 67 families being common to all three mutants (**Supplementary Figure S7**).

**Figure 3:**
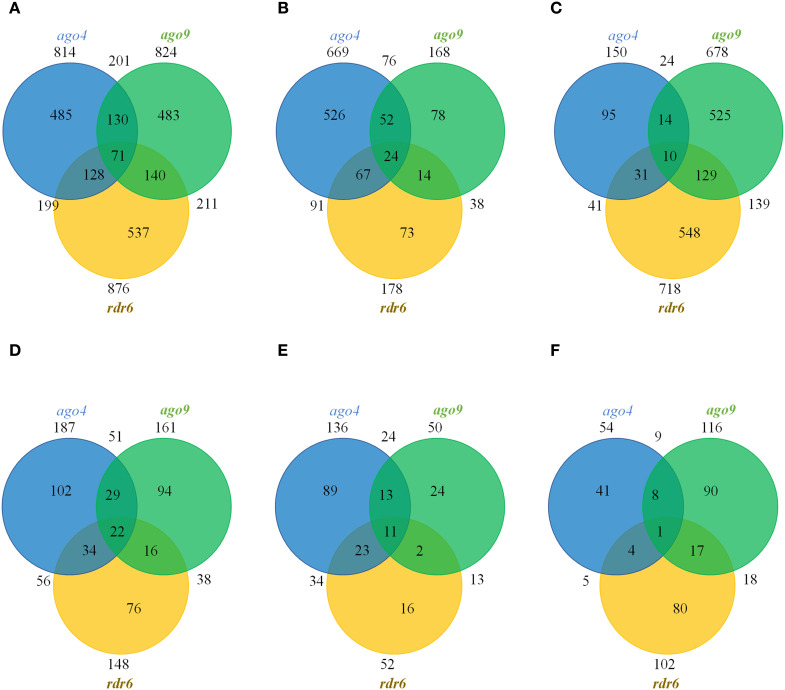
Venn distribution of TEs and genes mapping to DMRs in the CHH context. **(A)** Total number of DMRs containing TEs. **(B)** Total number of hypomethylated DMRs containing TEs. **(C)** Total number of hypermethylated DMRs containing TEs. **(D)** Total number of DMRs containing genes. **(E)** Total number of hypomethylated DMRs containing genes. **(F)** Total number of hypermethylated DMRs containing genes.

Of all the TEs targeted by DMRs, 59.58% (485/814), 58.62% (483/824), and 61.30% (537/876) were unique to *ago4*, *ago9*, and *rdr6*, respectively (**Figure 3A** and **Supplementary Table S6**). If only hypomethylated TEs are considered, 78.62% (526/669), 46.43% (78/168) and 41.01% (73/178) were unique to *ago4*, *ago9*, and *rdr6*, respectively, confirming the wider role that *ago4* appears to play in methylation of TEs during gynoecium development (**Figure 3B** **)**. If only *ago9* and *rdr6* were compared, only 20.5% (139/678) of DMRs map to the same TEs, confirming that targeted families of TEs are mostly distinct among the corresponding genes. This result is consistent with the fact that the families of hypermethylated TEs in both mutants were different (**Supplementary Figure S7**). Only 7.19% (10/139) of hypermethylated TEs are shared between *ago4*, *ago9*, and *rdr6* (**Figure 3C**), confirming the limited redundancy about their function during gynoecium development.

### Methylation at only a few genes is affected by mutations in *ago4*, *ago9* or *rdr6*

In all the three mutants a small fraction of DMRs map to gene elements, including their promoter region: 12.7% (204) in *ago4*, 12.4% (174) in *ago9*, and 10.3% (159) in *rdr6*. The total number of genes targeted by these DMRs are illustrated in **Figure 3D** and **Supplementary Table S7**. Only 22 genes had methylation patterns altered in all three mutants, opening possibilities for their functional investigation (**Figures 3D-F**). The identity of these 22 genes is presented in **Supplementary Table S8**, using annotation and description from TAIR10 (Berardini et al., 2015), complemented with UNIPROT information (UniProt Consortium, 2021) when necessary. Eight out of 22 genes were hypomethylated in all three mutant backgrounds: *ago4*, *ago9* and *rdr6* (At1g11785; At2g28400; At3g17750; At3g46470; At4g03380; At4g18010; At4g28397; At5g46295; **Supplementary Table S8**). These genes encode for two transmembrane proteins, the tyrosine kinase DYRKP-1, an RRM/RBD/RNP motif RNA-binding protein, a non-specific lipid-transfer-like protein, a protein with posphatase activity and two proteins of unknown function. A single gene encoding for a kinase-like protein (At3g23650) was hypermethylated in *ago4*, *ago9* and *rdr6* backgrounds. Of the remaining 13 genes, two were hypomethylated in *ago4* and *ago9*, but hypermethylated in *rdr6* (At1g52990 and At2g44175, encoding for a thioredoxin and acyl-CoA N-acyltransferase, respectively; **Supplementary Table S8**). Two are hypomethylated in *ago4* and *rdr6*, but hypermethylated in *ago9* (At1g18130 and At4g22810: encoding for a biotin synthetase and a protein AT-Hook nuclear localized protein, respectively; **Supplementary Table S8**). A single gene is hypermethylated in *ago4* and *ago9*, but hypomethylated in *rdr6* (At1g34410, encoding for the auxin response factor ARF21), or hypermethylated in *ago4* and *rdr6*, but hypomethylated in *ago9* (the hypothetical protein At4g03830; **Supplementary Table S8**). Four are hypomethylated in *ago4* but hypermethylated in *ago9* and *rdr6* (At2g18480; At3g02610; At4g15440; and At5g17600; encoding for the probable polyol transporter PLT3, the acyl-acyl carrier protein desaturase AAD2, the cytochrome CYP74B2, and the Arabidopsis toxic yeast protein ATL52, respectively; **Supplementary Table S8**). Finally, there are three genes that contain antagonistic but independent DMRs within their promoter or coding sequence: the hypothetical protein At2g39160 and the UMAMIT45 transporter (At3g28100) are hypomethylated in *ago4* and *rdr6* and contain two antagonistic RDMs in *ago9*; whereas the putative transmembrane protein DUF239 is hypomethylated *in ago4* but contains two antagonistic RDMs in *ago9* and *rdr6* (**Supplementary Table S8** **)**. Our results suggest that these genes are regulated by the RdDM pathway and could play unforeseen roles during female reproductive development.

To determine if these 22 genes were also included as RdDM targets in other organs or cells, we analyzed a collection of 9,993 DMR loci identified in leaves, plantlets and roots of *drm2* and *rdr2* mutants (Walker et al., 2018); and to a collection of 9,051 DMR loci identified in male meiocytes and pollen cells of *drm1 drm2* double mutants (Walker et al., 2018). As described in **Supplementary Table S9**, 15 out of these 22 genes (68.2%) are also included in RdDM-dependent DMRs of vegetative tissues. In addition, 18 out of 22 (81.8%) are included in RdDM-dependent DMRs of reproductive cells of the male lineage, suggesting that most of these genes are also regulated by RdDM during male gametogenesis (**Supplementary Table S9**). Only three out of 22 were not identified in RdDM -dependent DMRs of vegetative organs or male reproductive cells (At5g46295, At4g03830, At3g23650), suggesting that their regulation could be specific to female reproductive development (**Supplementary Table S9**). These results suggest that the RdDM-dependent regulation of these 22 genes is not restricted to reproductive tissues or specifically dependent on the activity of *AGO4*, *AGO9*, and *RDR6*, but rather shared by multiple genes involved in the RdDM pathway throughout Arabidopsis development.

## Discussion

The initiation of reproductive development in Arabidopsis entails the emergence from the shoot apical meristem of determined floral meristems that will progressively develop four whorls, including the central whorl of carpels that forms the gynoecium, which generally appears during stage 6 of flower primordium development (Herrera-Ubaldo and de Folter, 2022). In this study we provide the first compendium of genomic DNA methylation marks prevailing in the pre-meiotic gynoecium, allowing a comparison with other sporophytic and gametophytic methylomes in either wild-type or mutants of the RdDM pathway.

Surprisingly, our results show that the methylome of the Arabidopsis pre-meiotic gynoecium is characterized by TE and gene methylation levels that resemble those prevailing in male gametophytic cells rather than sporophytic tissues. By analyzing recently reported results (Zhou et al., 2022), we also found that methylation levels prevailing in differentiated ovules are also similar to those prevailing in the male meiocyte and pre-meiotic gynoecia. This tendency was already reflected in shoot stem cells, where methylation profiles of TEs in the CHH had also a tendency to resemble those of male meiocytes (Gutzat et al., 2020). Our results confirm this tendency at subsequent stages of female reproductive development, extending the results to genes in the CHH context, and TEs in the CG and CHG contexts. Although the methylome of the MMC has yet to be characterized, it is likely that the methylation profiles prevailing in the female meiocyte would be similar to those of the male meiocyte and pre-meiotic gynoecia. It is therefore possible to suggest that epigenetic reprogramming initiates in the shoot apical meristem, during the formation of the floral meristem, with methylation changes being progressively inherited to cells that subsequently give rise to the gynoecium and the ovule, closely resembling those that will ultimately prevail in gametophytic cells.

Cells of the male gametophytic lineage have similar levels of methylation, with higher levels of CG and CHG methylation in TEs, and lower levels of CHH methylation as compared to sporophytic organs (Walker et al., 2018). Given the similarity in methylation between the gynoecium and the male meiocyte, a similar developmental program could occur on the female reproductive side, with lower CHH methylation in the gynoecium and MMC than in the functional megaspore or the egg cell, for example. The molecular mechanism leading to decreased CHH methylation in the gynoecium remains to be determined. In the ovule of Arabidopsis, recent results have demonstrated that methylation levels depend on the CLASSY (CLSY) family of putative chromatin remodeling actors, specifically CLSY3 and CLSY4 (Zhou et al., 2022). Given the similarity in methylation levels between the ovule and gynoecium, it is possible that methylation levels in the pre-meiotic gynoecium will also depend on CLSY family members. Translational reporter fusions indicate that CLSY3 is highly expressed in the gynoecium and the ovule (Long et al., 2021). In contrast, its expression in the male reproductive side is confined to the tapetum cells, where CLSY3 is involved in the biogenesis of siRNAs that direct DNA methylation to meiocytes (Long et al., 2021).

On the other hand, the gynoecium methylation profiles of *ago4*, *ago9* and *rdr6* are virtually identical to those of wild type gynoecium in TEs and in genes, with only residual changes detected for TE and gene methylation levels in the CHH context, as expected for mutants involved in the RdDM pathway. The genome of *ago4* individuals contained the highest number of DMRs, confirming that AGO4 is likely the main effector of the canonical RdDM pathway in pre-meiotic gynoecia (Havecker et al., 2010; Cuerda-Gil and Slotkin, 2016). When compared to leaf methylomes, the frequency of DMRs in the CHH context was low for all three mutants. For comparison, homozygous individuals of *ago4-6* exhibited 3,731 and 1,606 DMRs in leaves and pre-meiotic gynoecia respectively, confirming that RdDM functional redundancy is particularly prevalent during female reproductive development (Havecker et al., 2010; Hernández-Lagana et al., 2016). No specific methylation defects had been characterized in *ago9* individuals during reproductive development, despite confirmation of its specific activity in the ovule (Olmedo-Monfil et al., 2010; She et al., 2013; Mendes et al., 2020). Our results offer the first compendium of genomic regions that are targeted for DNA methylation by AGO9 in pre-meiotic gynoecia. Although the role of *RDR6* in DNA methylation has been previously described (Nuthikattu et al., 2013; Stroud et al., 2013), our results represent the first collection of loci that show defects in RDR6-dependent DNA methylation.

The leaf methylome did not show hypermethylated DMRs in the genome of *ago9* individuals (Stroud et al., 2013), suggesting that its repressive role of DNA methylation might be specific to reproductive organs. Although *AGO4, AGO9* and *RDR6* participate in distinct variants of the RdDM pathway, our results show that these three genes generally act on distinct regions of the genome, as most of the corresponding DMRs targeting TEs or genes are specific to each of them. Nevertheless, we identified 22 genes that are targeted by all three genes in the pre-meiotic gynoecium, opening possibilities for their involvement in common reproductive phenotypes. Eleven of them are directly or indirectly related to mechanisms controlling reproductive development in Arabidopsis, either because of their phylogenetic relation with other family members playing a role in reproduction, or because of their putative interaction with previously characterized genes involved in different aspects of ovule or seed development (**Supplementary Table S10**). The subsequent functional analysis of these genes should determine of some of them are involved in gametic cell specification of other key developmental mechanism leading to the formation of the female gametophyte following meiosis and functional megaspore differentiation.

## Data availability statement

The datasets presentedin this study are deposited in the NCBI Gene Expression Omnibus(GEO), accession number GSE223459; https://www.ncbi.nlm.nih.gov/geo/query/acc.cgi?acc=GSE223459.

## Author contributions

J-PV-C, XF and QO-V designed the experiments. GL-M and CB-R provided expertise in sample collection and processing. XF, SD, BA, MV, EG-O, and QO-V provided computational support and analyzed data, QO-V and J-PV-C wrote the paper. All authors contributed to the article and approved the submitted version.

## Supplementary material

Supplementary Figure 1 Sampling of premeiotic gynoecia. **(A)** Gynoecia are collected using a stereo-microscope and dissecting ruler paper; the distance between two lines is 0.5 mm. **(B)** Premeiotic gynoecium of a wild-type *Col*-0 plant; premeiotic gynoecia from all three mutant alleles used in this study (*ago4-6, ago9-3, rdr6-15*) are phenotypically identical to wild-type. Scale bar = 10 μm

Supplementary Figure 2 Principal Component Analysis (PCA) of CG context reads from eight methylomes (two biological replicates per genotype).

Supplementary Figure 3 Comparison of methylation levels of TEs in the pre-meiotic of wild-type and RdDM mutants. The graph shows the methylation frequency shown at each nucleotide position for alignments in all three contexts for TEs and genes; wild-type (red), *ago4* (blue), *ago9* (green), *rdr6* (brown).

Supplementary Figure 4 Comparison of methylation levels of TEs and genes in the methylome of pre-meiotic gynoecia and differentiated ovules of Arabidopsis. The graph shows the methylation frequency shown at each nucleotide position for alignments in all three contexts; wild-type gynoecium (red), ovule (blue).

Supplementary Figure 5 Distribution of DMRs in the CHH context across all five Arabidopsis chromosomes. The density corresponds to the number of DMRs per 100 Kb.

Supplementary Figure 6 Most frequent superfamilies of TEs represented in hypo- or hypermethylated DMRs **(A)** Hypomethylated superfamilies. **(B)** Hypermethylated superfamilies.

Supplementary Figure 7 Most frequent families of TEs represented in DMRs. **(A)** Hypermethylated families. **(B)** Hypomethylated families.

Supplementary Table 1 Primers used for genotyping.

Supplementary Table 2 Global methylation levels in pre-meiotic gynoecia of wild-type, *ago4*, *ago9* and *rdr6* individuals.

Supplementary Table 3 DMRs distribution by size based in their sequence context

Supplementary Table 4 Distribution of DMRs by chromosome

Supplementary Table 5 Superfamilies of TEs represented in DMRs of *ago4*, *ago9* and *rdr6*

Supplementary Table 6 Transposable elements (TEs) represented in DMRs.

Supplementary Table 7 Genes represented in DMRs.

Supplementary Table 8 Genes mapping to DMRs in the CHH context of all the three mutants.

Supplementary Table 9 Genes included in DMRs of pre-meiotic gynoecia of *ago4*, *ago9*, and *rdr6* as well as in DMR RdDM targets of vegetative organs or male reproductive cells. ^a^ *soma*: DMRs from seedling, rosette leaf, cauline leaf and root methylomes in *drm2* and *rdr2* (from Walker et al., 2018); *sex*: DMRs from male meiocyte, microspore and sperm cell methylomes in the double mutant *drm1 drm2* (from Walker et al., 2018); *ago4*: DMRs from pre-meiotic gynoecia in *ago4-6* (this study); *ago9*: DMRs from pre-meiotic gynoecia in *ago9-3* (this study); *rdr6*: DMRs from pre-meiotic gynoecia in *rdr6-15* (this study).

Supplementary Table 10 Annotated information of 11 genes potentially having a reproductive function, and redundantly methylated by the common action of *AGO4*, *AGO9*, and *RDR6.*
