# Supplementary material for: Genomic methylation patterns in pre-meiotic gynoecia of wild-type and RdDM mutants of Arabidopsis
Source: Front Plant Sci. 2023 Mar 13;14:1123211. doi: 10.3389/fpls.2023.1123211 (PMC10040562; doi:10.3389/fpls.2023.1123211)
Supplement: Supplementary Figure 1 — Sampling of premeiotic gynoecia. (A) Gynoecia are collected using a stereo-microscope and dissecting ruler paper; the distance between two lines is 0.5 mm. (B) Premeiotic gynoecium of a wild-type Col-0 plant; premeiotic gynoecia from all three mutant alleles used in this study (ago4-6, ago9-3, rdr6-15) are phenotypically identical to wild-type. Scale bar = 10 μm [file DataSheet_1.docx]

**Supplementary Materials.**

**Supplementary Table S1. Primers used for genotyping.**

| **Gene** | **Primers name** | **Sequence (5’ to 3’)** | **Alignment Temperature** | **Size PCR product** | **Reference** |
| --- | --- | --- | --- | --- | --- |
| *AGO4* | *ago4-6_*LP  (Forward) | TTCTCCAGCTGGCTAGCTATG | 60°C | Wt= 1113bp  Mutant= 662bp | (Hernández-Lagana et al., 2016) |
|  | *ago4-6_*RP  (Reverse) | CCCAGAAAGGTGACATCTTTG |  |  | (Hernández-Lagana et al., 2016) |
| *AGO9* | *ago9-3_*LP  (Forward) | TGCAGGAACAATCATTGACAG | 61.5° | Wt= 1128bp  Mutant= 448bp | (Hernández-Lagana et al., 2016) |
|  | *ago9-3_*RP  (Reverse) | TCACGAAAAGAGCGAAATTTG |  |  | (Hernández-Lagana et al., 2016) |
| *RDR6* | *F_rdr6-15_*geno  (Forward) | ATGGGGTCAGAGGGAAATATGAA | 60°C | PCR product = 506bp  Wt dig. with MscI=506bp  Mutant dig. with MscI=396bp | ---- |
|  | *R_rdr6-15_*geno  (Reverse) | TTGCACGTGTTGTCAAAAGGATC |  |  |  |
| T-DNA | LBb1.3  (Forward) | ATTTTGCCGATTTCGGAAC |  | T-DNA of  SALK collection | --- |
| T-DNA | LB3  (Forward) | TAGCATCTGAATTTCATAACCAATCTCGATACAC |  | T-DNA of SAIL collection | --- |

**Supplementary Table S2. Global methylation levels in pre-meiotic gynoecia of wild-type, *ago4*, *ago9* and *rdr6* individuals.**

| Genotype | Sequence Context | Global methylation level (%) | Decrease of methylation (%) |
| --- | --- | --- | --- |
| Wt | CG | 22.37 | - |
|  | CHG | 8.63 | - |
|  | CHH | 1.14 | - |
| *ago4* | CG | 20.18 | 9.76 |
|  | CHG | 7.25 | 16.02 |
|  | CHH | 0.75 | 34.44 |
| *ago9* | CG | 18.93 | 15.36 |
|  | CHG | 6.40 | 25.80 |
|  | CHH | 0.68 | 40.70 |
| *rdr6* | CG | 17.16 | 23.27 |
|  | CHG | 5.81 | 32.70 |
|  | CHH | 0.74 | 35.17 |

**Supplementary Table S3. DMRs distribution by size based in their sequence context**

*See companion Excel file.*

**Supplementary Table S4. Distribution of DMRs by chromosome**

| **DMRs that belong to:** | **Sequence**  **Context** | **Chr 1** | **Chr 2** | **Chr 3** | **Chr 4** | **Chr 5** |
| --- | --- | --- | --- | --- | --- | --- |
| *ago4* | CG | 1140 | 752 | 891 | 663 | 970 |
|  | CHG | 548 | 444 | 494 | 411 | 498 |
|  | CHH | 424 | 260 | 330 | 242 | 350 |
|  | **Total** | **2,112** | **1,456** | **1,715** | **1,316** | **1,818** |
| *ago9* | CG | 651 | 373 | 409 | 290 | 614 |
|  | CHG | 261 | 314 | 288 | 230 | 295 |
|  | CHH | 376 | 228 | 283 | 225 | 290 |
|  | **Total** | **1,288** | **915** | **980** | **745** | **1,199** |
| *rdr6* | CG | 762 | 499 | 635 | 422 | 720 |
|  | CHG | 232 | 297 | 263 | 201 | 252 |
|  | CHH | 396 | 274 | 318 | 235 | 326 |
|  | **Total** | **1,390** | **1,070** | **1,216** | **858** | **1,298** |

**Supplementary Table S5. Superfamilies of TEs represented in DMRs of *ago4*, *ago9* and *rdr6***

*See companion Excel file*

**Supplementary Table S6. Transposable elements (TEs) represented in DMRs.**

*See companion Excel file*

**Supplementary Table S7. Genes represented in DMRs.**

*See companion Excel file*

**Supplementary Table S8. Genes mapping to DMRs in the CHH context of all the three mutants.**

| **Gene Id** | **TAIR Annotation** | **Comments** | **Methylation profile** |
| --- | --- | --- | --- |
| At1g11785 | Transmembrane protein | WEB1/PMI2-related (WPR) protein family | *ago4*(hypo) *ago9*(hypo) *rdr6*(hypo) |
| At2g28400 | Unknown protein | senescence regulator, could be related DUF584 family | *ago4*(hypo) *ago9*(hypo) *rdr6*(hypo) |
| At3g17750 | DYRKP-1 | Dual specificity tyrosine phosphoregulated kinase1 | *ago4*(hypo) *ago9*(hypo) *rdr6*(hypo) |
| At3g46470 | RNA-binding protein | RRM/RBD/RNP motif family protein | *ago4*(hypo) *ago9*(hypo) *rdr6*(hypo) |
| At4g03380 | Hypothetical protein | ---- | *ago4*(hypo) *ago9*(hypo) *rdr6*(hypo) |
| At4g18010 | 5PTASE2, AT5PTASE2 | Inositol(1,4,5)P3 5-Phosphatase II | *ago4*(hypo) *ago9*(hypo) *rdr6*(hypo) |
| At4g28397 | nsLTPs-like protein | Non-specific lipid-transfer-like protein | *ago4*(hypo) *ago9*(hypo) *rdr6*(hypo) |
| At5g46295 | Transmembrane protein | Interacts with bHLH DNA-binding protein | *ago4*(hypo) *ago9*(hypo) *rdr6*(hypo) |
| At2g39160 | Hypothetical protein | ---- | *ago4*(hypo) *ago9*(hypo/hyper) *rdr6*(hypo) |
| At3g28100 | UMAMIT45 | Usually Multiple Acids Move in and Out Transporter | *ago4*(hypo) *ago9*(hypo/hyper) *rdr6*(hypo) |

| At4g10210 | Putative transmembrane protein | DUF239 | *ago4*(hypo) *ago9*(hypo/hyper) *rdr6*(hypo/hyper) |
| --- | --- | --- | --- |
| At1g52990 | Thioredoxin (TRX) family protein | ---- | *ago4*(hypo) *ago9*(hypo) *rdr6*(hyper) |
| At2g44175 | Acyl-CoA N-acyltransferases (NAT) superfamily protein | ---- | *ago4*(hypo) *ago9*(hypo) *rdr6*(hyper) |
| At1g18130 | Biotin synthetase superfamily protein. | ---- | *ago4*(hypo) *ago9*(hyper) *rdr6*(hypo) |
| At4g22810 | AHL24 | AT-Hook nuclear localized protein 24 | *ago4*(hypo) *ago9*(hyper) *rdr6*(hypo) |
| At1g34410 | ARF21 | Auxin Response Factor 21 | *ago4*(hyper) *ago9*(hyper) *rdr6*(hypo) |
| At4g03830 | Hypothetical protein | ---- | *ago4*(hyper) *ago9*(hypo) *rdr6*(hyper) |
| At2g18480 | PLT3 | Probable polyol transporter 3 | *ago4*(hypo) *ago9*(hyper) *rdr6*(hyper) |
| At3g02610 | AAD2 | Acyl-acyl carrier protein desaturase | *ago4*(hypo) *ago9*(hyper) *rdr6*(hyper) |
| At4g15440 | CYP74B2 | CYP74B cytochrome p450 family member | *ago4*(hypo) *ago9*(hyper) *rdr6*(hyper) |
| At5g17600 | ATL52 | Arabidopsis Tóxicos en Levadura 52 | *ago4*(hypo) *ago9*(hyper) *rdr6*(hyper) |
| At3g23650 | Kinase-like protein | ---- | *ago4*(hyper) *ago9*(hyper) *rdr6*(hyper) |

**Supplementary Table S9. Genes included in DMRs of pre-meiotic gynoecia of *ago4*, *ago9*, and *rdr6* as well as in DMR RdDM targets of vegetative organs or male reproductive cells.**

| **ID** | **Gene body coordinates** | **Promoter coordinates** | **Mutant DMR origin^a^** | **DMR RdDM target coordinates** |
| --- | --- | --- | --- | --- |
| At1g11785 | 3,979,862 – 3,980,956 | 3,980,957 – 3,981,156 | *soma_1* | 3,979,952 – 3,981,001 |
| At1g11785 | 3,979,862 – 3,980,956 | 3,980,957 – 3,981,156 | *sex_1* | 3,980,102 – 3,981,051 |
| At1g11785 | 3,979,862 – 3,980,956 | 3,980,957 – 3,981,156 | *ago4_1* | 3,980,700 – 3,980,950 |
| At1g11785 | 3,979,862 – 3,980,956 | 3,980,957 – 3,981,156 | *ago9_1* | 3,980,800 – 3,980,900 |
| At1g11785 | 3,979,862 – 3,980,956 | 3,980,957 – 3,981,156 | *rdr6_1* | 3,980,800 – 3,980,950 |
| At2g28400 | 12,150,380 – 12,150,502 | 12,150,180 – 12,150,379 | *sex_2* | 12,149,874 – 12,150,823 |
| At2g28400 | 12,150,380 – 12,150,502 | 12,150,180 – 12,150,379 | *ago4_2* | 12,150,300 – 12,150,400 |
| At2g28400 | 12,150,380 – 12,150,502 | 12,150,180 – 12,150,379 | *ago9_2* | 12,150,350 – 12,150,450 |
| At2g28400 | 12,150,380 – 12,150,502 | 12,150,180 – 12,150,379 | *rdr6_2* | 12,150,300 – 12,150,450 |
| At3g17750 | 6,073,769 – 6,078,722 | 6,073,569 – 6,073,768 | *soma_2* | 6,073,295 – 6,073,744 |
| At3g17750 | 6,073,769 – 6,078,722 | 6,073,569 – 6,073,768 | *sex_3* | 6,073,245 – 6,073,744 |
| At3g17750 | 6,073,769 – 6,078,722 | 6,073,569 – 6,073,768 | *ago4_3* | 6,073,400 – 6,073,750 |
| At3g17750 | 6,073,769 – 6,078,722 | 6,073,569 – 6,073,768 | *ago9_3* | 6,073,550 – 6,073,750 |
| At3g17750 | 6,073,769 – 6,078,722 | 6,073,569 – 6,073,768 | *rdr6_3* | 6,073,400 – 6,073,750 |
| At3g46470 | 17,099,916 – 17,100,973 | 17,100,974 – 17,101,173 | *soma_3* | 17,101,116 – 17,101,465 |
| At3g46470 | 17,099,916 – 17,100,973 | 17,100,974 – 17,101,173 | *sex_4* | 17,100,916 – 17,102,015 |
| At3g46470 | 17,099,916 – 17,100,973 | 17,100,974 – 17,101,173 | *ago4_4* | 17,101,000 – 17,101,100 |
| At3g46470 | 17,099,916 – 17,100,973 | 17,100,974 – 17,101,173 | *ago9_4* | 17,100,900 – 17,101,050 |
| At3g46470 | 17,099,916 – 17,100,973 | 17,100,974 – 17,101,173 | *rdr6_4* | 17,100,950 – 17,101,100 |
| At4g03380 | 1,485,049 – 1,490,228 | 1,490,229 – 1,490,428 | *soma_4* | 1,488,352 – 1,488,601 |
| At4g03380 | 1,485,049 – 1,490,228 | 1,490,229 – 1,490,428 | *sex_5* | 1,486,452 – 1,487,601 |
| At4g03380 | 1,485,049 – 1,490,228 | 1,490,229 – 1,490,428 | *ago4_5* | 1,486,500 – 1,486,650 |
| At4g03380 | 1,485,049 – 1,490,228 | 1,490,229 – 1,490,428 | *ago9_5* | 1,486,500 – 1,486,650 |
| At4g03380 | 1,485,049 – 1,490,228 | 1,490,229 – 1,490,428 | *rdr6_5* | 1,485,100 – 1,485,200 |
| At4g18010 | 9,989,753 – 9,990,136 | 9,990,137 – 9,990,336 | *sex_6* | 9,988,889 – 9,989,938 |
| At4g18010 | 9,989,753 – 9,990,136 | 9,990,137 – 9,990,336 | *ago4_6* | 9,989,750 – 9,989,850 |
| At4g18010 | 9,989,753 – 9,990,136 | 9,990,137 – 9,990,336 | *ago9_6* | 9,989,750 – 9,989,850 |
| At4g18010 | 9,989,753 – 9,990,136 | 9,990,137 – 9,990,336 | *rdr6_6* | 9,989,750 – 9,989,850 |
| At4g28397 | 14,045,924 – 14,046,689 | 14,045,724 – 14,045,923 | *soma_5* | 14,045,545 – 14,045,844 |
| At4g28397 | 14,045,924 – 14,046,689 | 14,045,724 – 14,045,923 | *sex_7* | 14,045,495 -14,045,794 |
| At4g28397 | 14,045,924 – 14,046,689 | 14,045,724 – 14,045,923 | *ago4_7* | 14,045,650 – 14,045,800 |
| At4g28397 | 14,045,924 – 14,046,689 | 14,045,724 – 14,045,923 | *ago9_7* | 14,045,650 – 14,045,800 |
| At4g28397 | 14,045,924 – 14,046,689 | 14,045,724 – 14,045,923 | *rdr6_7* | 14,045,650 – 14,045,800 |
| At5g46295 | 18,779,796 – 18,780,205 | 18,780,206 – 18,780,405 | *ago4_8* | 18,780,300 – 18,780,400 |
| At5g46295 | 18,779,796 – 18,780,205 | 18,780,206 – 18,780,405 | *ago9_8* | 18,780,300 – 18,780,400 |
| At5g46295 | 18,779,796 – 18,780,205 | 18,780,206 – 18,780,405 | *rdr6_8* | 18,780,300 -18,780,400 |
| At2g39160 | 16,333,490 – 16,334,173 | 16,334,174 – 16,334,373 | *sex_8* | 16,333,323 – 16,333,622 |
| At2g39160 | 16,333,490 – 16,334,173 | 16,334,174 – 16,334,373 | *ago4_9* | 16,333,500 – 16,333,850 |
| At2g39160 | 16,333,490 – 16,334,173 | 16,334,174 – 16,334,373 | *ago9_9* | 16,333,600 – 16,333,900 |
| At2g39160 | 16,333,490 – 16,334,173 | 16,334,174 – 16,334,373 | *rdr6_9* | 16,333,500 – 16,333,650 |
| At3g28100 | 10,456,099 – 10,460,965 | 10,455,899 – 10,456,098 | *soma_6* | 10,457,364 – 10,458,363 |
| At3g28100 | 10,456,099 – 10,460,965 | 10,455,899 – 10,456,098 | *sex_9* | 10,457,464 – 10,458,363 |
| At3g28100 | 10,456,099 – 10,460,965 | 10,455,899 – 10,456,098 | *ago4_10* | 10,459,800 – 10,459,900 |
| At3g28100 | 10,456,099 – 10,460,965 | 10,455,899 – 10,456,098 | *ago9_10* | 10,457,450 – 10,457,600 |
| At3g28100 | 10,456,099 – 10,460,965 | 10,455,899 – 10,456,098 | *rdr6_10* | 10,457,500 – 10,457,600 |
| At4g10210 | 6,357,052 – 6,360,377 | 6,356,852 – 6,357,051 | *soma_7* | 6,359,755 – 6,359,954 |
| At4g10210 | 6,357,052 – 6,360,377 | 6,356,852 – 6,357,051 | *sex_10* | 6,356,905 – 6,357,954 |
| At4g10210 | 6,357,052 – 6,360,377 | 6,356,852 – 6,357,051 | *ago4_11* | 6,357,300 – 6,357,400 |
| At4g10210 | 6,357,052 – 6,360,377 | 6,356,852 – 6,357,051 | *ago9_11* | 6,357,250 – 6,357,400 |
| At4g10210 | 6,357,052 – 6,360,377 | 6,356,852 – 6,357,051 | *rdr6_11* | 6,357,550 – 6,357,650 |
| At1g52990 | 19,740,503 – 19,743,449 | 19,743,450 – 19,743,649 | *soma_8* | 19,741,483 – 19,742,182 |
| At1g52990 | 19,740,503 – 19,743,449 | 19,743,450 – 19,743,649 | *sex_11* | 19,741,533 – 19,742,182 |
| At1g52990 | 19,740,503 – 19,743,449 | 19,743,450 – 19,743,649 | *ago4_12* | 19,742,050 – 19,742,150 |
| At1g52990 | 19,740,503 – 19,743,449 | 19,743,450 – 19,743,649 | *ago9_12* | 19,742,050 – 19,742,150 |
| At1g52990 | 19,740,503 – 19,743,449 | 19,743,450 – 19,743,649 | *rdr6_12* | 19,741,850 – 19,742,050 |
| At2g44175 | 18,268,788 – 18,269,214 | 18,268,588 – 18,268,787 | *sex_12* | 18,267,825 – 18,268,674 |
| At2g44175 | 18,268,788 – 18,269,214 | 18,268,588 – 18,268,787 | *ago4_13* | 18,269,050 – 18,269,150 |
| At2g44175 | 18,268,788 – 18,269,214 | 18,268,588 – 18,268,787 | *ago9_13* | 18,268,700 – 18,268,800 |
| At2g44175 | 18,268,788 – 18,269,214 | 18,268,588 – 18,268,787 | *rdr6_13* | 18,268,600 – 18,268,950 |
| At1g18130 | 6,235,895 – 6,238,799 | 6,235,695 – 6,235,894 | *soma_9* | 6,237,308 – 6,237,757 |
| At1g18130 | 6,235,895 – 6,238,799 | 6,235,695 – 6,235,894 | *sex_13* | 6,236,558 – 6,237,857 |
| At1g18130 | 6,235,895 – 6,238,799 | 6,235,695 – 6,235,894 | *ago4_14* | 6,237,650 – 6,237,750 |
| At1g18130 | 6,235,895 – 6,238,799 | 6,235,695 – 6,235,894 | *ago9_14* | 6,237,650 – 6,237,750 |
| At1g18130 | 6,235,895 – 6,238,799 | 6,235,695 – 6,235,894 | *rdr6_14* | 6,236,900 – 6,237,000 |
| At4g22810 | 11,978,005 – 11,983,071 | 11,983,072 – 11,983,271 | *soma_10* | 11,979,090 – 11,979,539 |
| At4g22810 | 11,978,005 – 11,983,071 | 11,983,072 – 11,983,271 | *sex_14* | 11,978,890 – 11,979,539 |
| At4g22810 | 11,978,005 – 11,983,071 | 11,983,072 – 11,983,271 | *ago4_15* | 11,979,500 – 11,979,600 |
| At4g22810 | 11,978,005 – 11,983,071 | 11,983,072 – 11,983,271 | *ago9_15* | 11,978,750 – 11,979,000 |
| At4g22810 | 11,978,005 – 11,983,071 | 11,983,072 – 11,983,271 | *rdr6_15* | 11,979,500 – 11,979,600 |
| At1g34410 | 12,577,722 – 12,580,824 | 12,577,522 – 12,577,721 | *soma_11* | 12,577,623 – 12,578,172 |
| At1g34410 | 12,577,722 – 12,580,824 | 12,577,522 – 12,577,721 | *ago4_16* | 12,578,000 – 12,578,150 |
| At1g34410 | 12,577,722 – 12,580,824 | 12,577,522 – 12,577,721 | *ago9_16* | 12,577,700 – 12,577,950 |
| At1g34410 | 12,577,722 – 12,580,824 | 12,577,522 – 12,577,721 | *rdr6_16* | 12,578,000 – 12,578,100 |
| At4g03830 | 1,790,440 – 1,792,458 | 1,790,240 – 1,790,439 | *ago4_17* | 1,790,750 – 1,790,850 |
| At4g03830 | 1,790,440 – 1,792,458 | 1,790,240 – 1,790,439 | *ago9_17* | 1,791,550 – 1,791,700 |
| At4g03830 | 1,790,440 – 1,792,458 | 1,790,240 – 1,790,439 | *rdr6_17* | 1,791,750 – 1,791,850 |
| At2g18480 | 8,009,323 – 8,011,255 | 8,011,256 – 8,011,455 | *soma_12* | 8,009,119 – 8,009,718 |
| At2g18480 | 8,009,323 – 8,011,255 | 8,011,256 – 8,011,455 | *sex_15* | 8,009,419 – 8,009,618 |
| At2g18480 | 8,009,323 – 8,011,255 | 8,011,256 – 8,011,455 | *ago4_18* | 8,009,450 – 8,009,600 |
| At2g18480 | 8,009,323 – 8,011,255 | 8,011,256 – 8,011,455 | *ago9_18* | 8,009,300 – 8,009,400 |
| At2g18480 | 8,009,323 – 8,011,255 | 8,011,256 – 8,011,455 | *rdr6_18* | 8,009,300 – 8,009,400 |
| At3g02610 | 555,626 – 557,723 | 555,426 – 555,625 | *soma_13* | 557,494 – 558,143 |
| At3g02610 | 555,626 – 557,723 | 555,426 – 555,625 | *sex_16* | 557,494 – 557,993 |
| At3g02610 | 555,626 – 557,723 | 555,426 – 555,625 | *ago4_19* | 557,500 – 557,750 |
| At3g02610 | 555,626 – 557,723 | 555,426 – 555,625 | *ago9_19* | 557,650 – 557,750 |
| At3g02610 | 555,626 – 557,723 | 555,426 – 555,625 | *rdr6_19* | 557,500 – 557,600 |
| At4g15440 | 8,835,495 – 8,838,676 | 8,835,295 – 8,835,494 | *soma_14* | 8,836,502 – 8,837,151 |
| At4g15440 | 8,835,495 – 8,838,676 | 8,835,295 – 8,835,494 | *sex_17* | 8,836,452 – 8,837,251 |
| At4g15440 | 8,835,495 – 8,838,676 | 8,835,295 – 8,835,494 | *ago4_20* | 8,837,050 – 8,837,150 |
| At4g15440 | 8,835,495 – 8,838,676 | 8,835,295 – 8,835,494 | *ago9_20* | 8,837,000 – 8,837,150 |
| At4g15440 | 8,835,495 – 8,838,676 | 8,835,295 – 8,835,494 | *rdr6_20* | 6,357,052 – 6,360,377 |
| At5g17600 | 5,799,905 – 5,801,244 | 5,801,245 – 5,801,444 | *soma_15* | 5,801,249 – 5,802,098 |
| At5g17600 | 5,799,905 – 5,801,244 | 5,801,245 – 5,801,444 | *sex_18* | 5,801,249 – 5,802,148 |
| At5g17600 | 5,799,905 – 5,801,244 | 5,801,245 – 5,801,444 | *ago4_21* | 5,801,350 – 5,801,500 |
| At5g17600 | 5,799,905 – 5,801,244 | 5,801,245 – 5,801,444 | *ago9_21* | 5,801,350 – 5,801,450 |
| At5g17600 | 5,799,905 – 5,801,244 | 5,801,245 – 5,801,444 | *rdr6_21* | 5,801,250 – 5,801,350 |
| At3g23650 | 8,510,689 – 8,511,196 | 8,511,197 – 8,511,396 | *ago4_22* | 8,511,150 – 8,511,250 |
| At3g23650 | 8,510,689 – 8,511,196 | 8,511,197 – 8,511,396 | *ago9_22* | 8,511,100 – 8,511,250 |
| At3g23650 | 8,510,689 – 8,511,196 | 8,511,197 – 8,511,396 | *rdr6_22* | 8,510,800 – 8,510,900 |

^a^ *soma*: DMRs from seedling, rosette leaf, cauline leaf and root methylomes in *drm2* and *rdr2* (from Walker et al., 2018); *sex*: DMRs from male meiocyte, microspore and sperm cell methylomes in the double mutant *drm1 drm2* (from Walker et al., 2018); *ago4*: DMRs from pre-meiotic gynoecia in *ago4-6* (this study); *ago9*: DMRs from pre-meiotic gynoecia in *ago9-3* (this study); *rdr6*: DMRs from pre-meiotic gynoecia in *rdr6-15* (this study).

**Supplementary Table S10. Annotated information of 11 genes potentially having a reproductive function, and redundantly methylated by the common action of *AGO4*, *AGO9*, and *RDR6.***

1. **At1g34410** (*ARF21, AUXIN RESPONSE FACTOR 21).* Overexpression of an RNAi fragment targeted against its group of highly similar ARFs (ARF12-15 and ARF20-23) caused embryonic defects. *ARF12*-15 and *ARF20*-22 showed expression in the embryo surrounding endosperm (Lokerse et al., 2011). The expansion of *ARF3* expression into lateral epidermal cells of ovule primordia in a TAS3 ta-siRNA-insensitive mutant led to the formation of multiple MMC-like cells (Su et al., 2017), also *ARF3* is involved in gynoecium patterning (Heisler et al., 2001)
2. **At3g02610** (*AAD2*, *ACYL-ACYL CARRIER PROTEIN DESATURASE (AAD) 2)*, encodes one of two ∆9 palmitoyl-ACP desaturases responsible for the biosynthesis of ω-7 fatty acids in the maturing endosperm (Bryant et al., 2016).
3. **At5g17600** (*ATL52,* *Arabidopsis Tóxicos en Levadura (ATL) 52*). *ATL52* is expressed in Arabidopsis flowers (Hu et al., 2003). Physical interaction: At5g65430 *GENERAL REGULATORY FACTOR 8* (*GRF8*) member of the 14-3-3 proteins that is reported to interact with the *BZR1* transcription factor involved in brassinosteroid (BR) signaling and may affect the nucleocytoplasmic shuttling of *BZR1* that play a central role in BR signal transduction. BR-insensitive mutants generate supernumerary MMCs (Cai et al., 2022).

**4) At4g15440** (*CYP74B2, member of the CYP74B cytochrome p450 family, HPL1, HYDROPEROXIDE LYASE 1*)**.**  In the ecotype Columbia (Col) the *CYP74B2* gene contains a 10-nucleotide deletion in its first exon that causes it to code for a protein not containing the P450 signature typical of other CYP74B subfamily members and show reduced transcript levels (Duan et al., 2005). CYP74B2 transcripts were compared in leaves and flowers of the Columbia, Landsberg *erecta* (L*er*), and Wassilewskija (Ws) ecotypes using semiquantitative RT-PCR blot assays. Accumulation of CYP74B2 transcripts in Col leaves and flowers are 2.4- to 5.2-fold lower than in L*er* leaves and flowers and 4.7-fold lower than in Ws leaves (Duan et al., 2005).

The CYP74s family present two subfamilies in Arabidopsis: *CYP74A* and *CYP74B*, which seem to be present in most, if not all, plant species, each have a single member in the Arabidopsis genome: *CYP74A1* and *CYP74B2*. Expression of *CYP74A1* is predominantly found in leaves and flowers of mature Arabidopsis plants. *CYP74A1* transient expression is also observed in flower organs: weak and transient in developing carpels, very strong in maturing pollen and at the basis of anther filaments (Bak et al., 2011).

*CYP85A2* can synthesize the most bioactive brassinosteroid, brassinolide and *cyp85a2* exhibiting a weakly dwarfed phenotype (Bak et al., 2011) but during the ovule development *cyp85a2* plants displayed supernumerary enlarged MMC-like cells (Cai et al., 2022)

**5) At3g28100** (*UMAMIT45*, *USUALLY MULTIPLE ACIDS MOVE IN AND OUT TRANSPORTERS 45*) Nodulin MtN21-like transporter family protein. *UMAMIT45* is grouped in the same subfamily with other seven genes: AT3G28050 (*UMAMIT41*, pUMAMIT41: UMAMIT41-GFP was expressed in the seed and silique), AT3G28070 (*UMAMIT46*), AT3G28080 (*UMAMIT47*), AT3G28130 (*UMAMIT44*), AT5G40210 (*UMAMIT42*), At5g40230 (*UMAMIT37*, pUMAMIT37:UMAMIT37-GFP was transiently expressed at the unloading zone of the seed), AT5G40240 (*UMAMIT40*) (Ladwig et a.l, 2012; Doktorarbeit, 2016). Member of other subfamily that belongs to the same gene family AT1G44800 (*UMAMIT18/SIAR1*), may be required for releasing amino acids from the phloem to the apoplasmic space, making them subsequently available for import into adjacent cells of the endosperm and the embryo (Ladwig et a.l, 2012)

**6) At2g44175** (Acyl-CoA N-acyltransferases (NAT) superfamily protein). At2g44175 displays a high level of identity to both *Myristoyl-CoA, N-myristoyltransferase1 ( NMT1)* (53%) and *NMT2* (At2g44170) (48%). The ORF preceding *NMT2* is At2g44175, that encodes a 113–amino acid protein but no cDNA corresponding to this ORF has yet been found (Pierre et al., 2007). Strong *NMT1* expression appeared to be associated with the metabolically active areas of vegetative organs, such as developing leaves and root apical and lateral meristems. In flowers, the *NMT1* promoter was found to be highly active in pollen grains, within stamens, or in female areas. *NMT1* mutant displays severe SAM Defects on the late embryonic development (Pierre et al., 2007). N-Myristoylation (MYR) is required for embryonic development in animals and plants (Pierre et al., 2007).

**7) At4g22810** (*AHL24*, *AT-HOOK MOTIF NUCLEAR LOCALIZED PROTEIN 24)*. Transcription factor that specifically binds AT-rich DNA sequences related to the nuclear matrix attachment regions (MARs). The Arabidopsis genome contains 29 genes encoding the AT-hook motif DNA-binding protein (AHL). AHL genes are important during zygotic embryogenesis and modulates hypocotyl growth redundantly by interacting with each (Karami et al., 2021; Zhao et al., 2013). The closest *AHL24* paralogs are At4g12050 (*AHL26*), AT2G45430 (*AHL22*) and At3g60870 (*AHL18*) (Karami et al., 2021). *AHL22* overexpression results in delayed flowering. It is likely to act redundantly with *AHL18*, *AHL27* and *AHL29* in the regulation of flowering (TAIR10).

Overexpression of some Arabidopsis AHL genes (*AHL15*, *AHL19*, *AHL20*, and *AHL29*) did induce somatic embryogenesis (Karami et al., 2021).

*AHL24* is coexpressed with *AHL22*, *AHL26, AHL21* or *GIANT KILLER* that is a direct target of AGAMOUS (AG), and AG is involved in regulates patterning and differentiation of reproductive organs. Also, *AHL24* is coexpressed with *ATBS1* (*ACTIVATION-TAGGED BRI1(BRASSINOSTEROID-INSENSITIVE 1)-SUPPRESSOR 1*, At1g74500) that plays a role in brassinosteroid signaling.

**8) At2g18480** (*PLT3*, *Probable polyol transporter 3*) (Klepek et al., 2005). Analysis of a phylogenetic tree shows that Arabidopsis *PLT3*, *PLT5* (At3g18830), and *PLT6* (At4g36670) show the highest degree of sequence conservation, share ∼70 to 80% identity (Klepek et al., 2005). RT-PCR analyses and *PLT5* promoter-reporter gene revealed that PLT5 is most strongly expressed in Arabidopsis roots, but also in the vascular tissue of leaves and in vascular strands of the sepals and in the ovary (Klepek et al., 2005).

*PLT3* has physical interaction with: *CYCLIC NUCLEOTIDE-GATED CHANNEL 18* (*GNGC18, A*T5G14870) that is asymmetrically localized to the plasma membrane at the growing tip of the pollen tube and is involved in pollen tube growth and pollen tube guidance to ovules. Also, *PLT3* has physical interaction with *MILDEW RESISTANCE LOCUS O 4* (*MLO4,* AT1G11000) gene that is expressed during early seedling growth, in roots and lateral root primordia, in flower and fruit abscission zone.

**9) At1g11785** (transmembrane protein). High BlastP homology with *WEB1/PMI2-related-a1 (WPRa1)* AT1G12150 (Blastp using key domain in aminoacids 28 – 45: Query cover 100%, E-value 1e-10, Percent Identity 100%) that is described as weak chloroplast movement under blue light protein, DUF827. The public microarray database Genevestigator revealed that WPR genes were ubiquitously expressed, although many WPR genes showed some tissue specific expression pattern. *WPRa1* had very high relative expression intensity in pollen compared to other tissues, based on microarray data (Kodama et al., 2011).

**10) At4g28397** (non-specific lipid-transfer-like protein (nsLTPs-like)). In Arabidopsis non-specific lipid-transfer proteins (nsLTPs) are also known to play important roles in sexual reproduction (Liu et al., 2015). Member of nsLTPs family, *LIPID TRANSFER PROTEIN5* (*LTP5*) functions in pollen tube guidance in fertilization, LTP5 expression levels were very low in the style, petals, and sepals and only with a 5 d GUS reaction was it possible to show LTP5 gene expression in pollen and the transmitting tract (TT) in the pistil, LTP1 was expressed in almost all floral tissues, with the highest level in the stigma and the style, *LTP3* in the ovules and the sepals, *LTP6* in the style, the ovules, anther filaments, and the sepals (Chae et al., 2010).

High homology with *Arabidopsis thaliana* *anther 7* (*ATA7)* also named *AtLtpY.4* (AT4G28395) (Blastp using all aminoacid sequence: Query cover: 100%; E value:2e-60; Percent identity : 90.09%; Blastp using peptide signal sequence: Query cover: 100%; E value:7e-7; Percent identity : 100%). *ATA7* encodes a nsLTP gene identified in an expression studie of novel anther- specific genes of Arabidopsis*,* reported to be specifically transcribed in the tapetal cell layer in inflorescences of wild-type (Boutrot et al., 2008; Rubinelli et al., 1998)..

**11) At3g17750** (*DYRKP-1*, *PLANT-SPECIFIC DUAL-SPECIFICITY TYROSINE PHOSPHORYLATION-REGULATED KINASE 1*)**.** DUAL-SPECIFICITY TYROSINE PHOSPHORYLATION-REGULATED KINASES (DYRKs) in yeast and mammals are well known as key regulators of the cell cycle and differentiation (Furuya et al., 2021). DYRKP subgroup is plant-specific (Iwabuchi et al., 2019). In Marchantia polymorpha the sole DYRKP ortholog mutant exhibited abnormal, shrunken morphologies with less flattening in their vegetative plant bodies, thalli, and male reproductive organs, antheridial receptacles (Furuya et al., 2021).

*DYRKP-1* is co-expressed with *UBIQUITIN-SPECIFIC PROTEASE 26* (*UBP26)* that encodes a ubiquitin-specific protease which catalyzes deubiquitination of histone H2B and is required for heterochromatin silencing. *UBP26* loss of function mutations display autonomous endosperm development and embryo arrest. *UBP26* loss of function also results in an increase in expression of the PcG complex target gene *PHERES1*. *DYRKP-1* is co-expressed with At1g08060 (*MOM*, *MAINTENANCE OF METHYLATION*, *MOM1*, *MORPHEUS MOLECULE 1*) that is involved in a DNA methylation-independent epigenetic silencing pathway that has high expression levels in sperm cells (Borges et al., 2008)

| A)  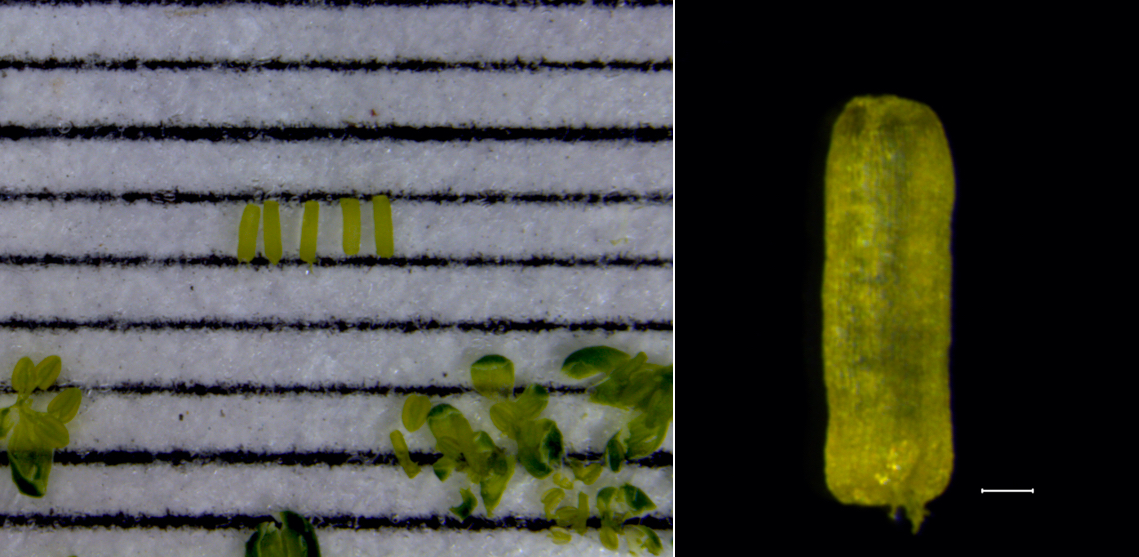 | B)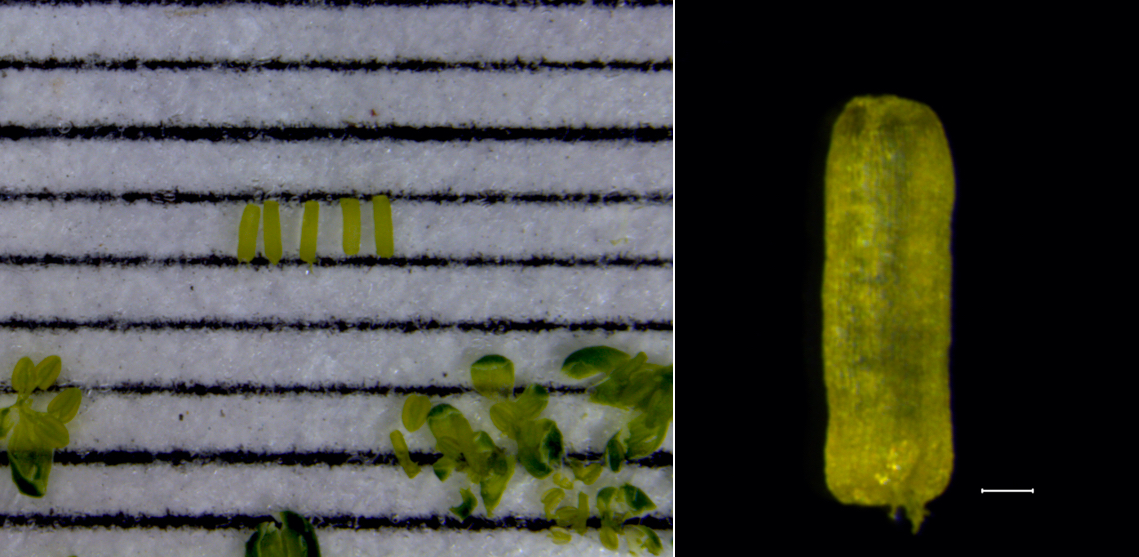 |
| --- | --- |

**Supplementary** Figure S1. Sampling of premeiotic gynoecia. (A) Gynoecia are collected using a stereo-microscope and dissecting ruler paper; the distance between two lines is 0.5 mm. (B) Premeiotic gynoecium of a wild-type *Col*-0 plant; premeiotic gynoecia from all three mutant alleles used in this study (*ago4-6, ago9-3, rdr6-15*) are phenotypically identical to wild-type. Scale bar = 10 μm

**Supplementary** Figure S2. **Principal Component Analysis (PCA) of CG context reads from eight methylomes (two biological replicates per genotype).**

| TEs_CG   | Genes_CG   |
| --- | --- |
| TEs_CHG   | Genes_CHG   |
| TEs_CHH   | Genes_CHH   |

**Supplementary** Figure S3. **Comparison of methylation levels of TEs in the pre-meiotic of wild-type and RdDM mutants.** The graph shows the methylation frequency shown at each nucleotide position for alignments in all three contexts for TEs and genes; wild-type (red), *ago4* (blue), *ago9* (green), *rdr6* (brown).

| TEs_CG | Genes_CG  |
| --- | --- |
| TEs_CHG | Genes_CHG  |
| TEs_CHH   | Genes_CHH   |

**Supplementary** Figure S4. **Comparison of methylation levels of TEs and genes in the methylome of pre-meiotic gynoecia and differentiated ovules of Arabidopsis.** The graph shows the methylation frequency shown at each nucleotide position for alignments in all three contexts; wild-type gynoecium (red), ovule (blue).

|  |  |  |  |  |
| --- | --- | --- | --- | --- |
|  |  |  |  |  |
|  |  |  |  |  |

**Supplementary** Figure S5. Distribution of DMRs in the CHH context across all five Arabidopsis chromosomes. The density corresponds to the number of DMRs per 100 Kb.

| A)   |
| --- |
| B)   |

**Supplementary** Figure S6. Most frequent superfamilies of TEs represented in hypo- or hypermethylated DMRs (A) Hypomethylated superfamilies. (B) Hypermethylated superfamilies.

| **A)**   | **B)**  **** |
| --- | --- |

**Supplementary** Figure S7. **Most frequent families of TEs represented in DMRs**. (A) Hypermethylated families. (B) Hypomethylated families.
